# Non-patient related variables affecting levels of vascular endothelial growth factor in urine biospecimens

**DOI:** 10.1111/j.1582-4934.2008.00182.x

**Published:** 2008-08-11

**Authors:** M J Kirk, R M Hayward, M Sproull, T Scott, S Smith, T Cooley-Zgela, N S Crouse, D E Citrin, K Camphausen

**Affiliations:** Radiation Oncology Branch, Center for Cancer Research, National Cancer Institute, National Institutes of Health, Department of Health and Human ServicesBethesda, MD, USA

**Keywords:** angiogenesis, VEGF, tumour markers, urine, biospecimens

## Abstract

Vascular endothelial growth factor (VEGF) is an angiogenic protein proposed to be an important biomarker for the prediction of tumour growth and disease progression. Recent studies suggest that VEGF measurements in biospecimens, including urine, may have predictive value across a range of cancers. However, the reproducibility and reliability of urinary VEGF measurements have not been determined. We collected urine samples from patients receiving radiation treatment for glioblastoma multiforme (GBM) and examined the effects of five variables on measured VEGF levels using an ELISA assay. To quantify the factors affecting the precision of the assay, two variables were examined: the variation between ELISA kits with different lot numbers and the variation between different technicians. Three variables were tested for their effects on measured VEGF concentration: the time the specimen spent at room temperature prior to assay, the addition of protease inhibitors prior to specimen storage and the alteration of urinary pH. This study found that VEGF levels were consistent across three different ELISA kit lot numbers. However, significant variation was observed between results obtained by different technicians. VEGF concentrations were dependent on time at room temperature before measurement, with higher values observed 3–7 hrs after removal from the freezer. No significant difference was observed in VEGF levels with the addition of protease inhibitors, and alteration of urinary pH did not significantly affect VEGF measurements. In conclusion, this determination of the conditions necessary to reliably measure urinary VEGF levels will be useful for future studies related to protein biomarkers and disease progression.

## Introduction

Evidence for the role of angiogenesis in cancer biology was first suggested by Judah Folkman, who found that solid tumours remained dormant and limited in size in the absence of neovascu-larization [1]. This observation has driven further research directed at targeting angiogenesis as a means of halting tumour growth, which has resulted in the current therapeutic use of several angio-genesis inhibitors as anticancer agents [2–6]. Recently, several studies have investigated the quantification of angiogenic proteins in urine for use in cancer diagnosis and prognosis [7–10]. Vascular endothelial growth factor (VEGF) is a ubiquitous angio-genic protein that acts as a mitogenic stimulus on endothelial cells. Secretion of VEGF by tumour cells can be detected in various body fluids including blood, urine and saliva [11–14]. If urinary levels of VEGF can be accurately quantified, evaluation of this protein may potentially provide a convenient and non-invasive predictor of tumour behaviour and the overall angiogenic state of the host. However, as with any marker evaluated in biological specimens, the stability of VEGF in the urine between the time of sample collection and analysis is of concern. Often, initial studies of a marker report promising results but subsequent confirmatory studies of the same candidate marker conflict, potentially due to the use of unstandardized methods that lack reproducibility [15]. Few studies have investigated the role of variables that could potentially affect VEGF measurements in urine [16]. In this study, we sought to examine several variables that may affect the biomol-ecular profile of urine specimens. Whereas Hayward *et al.* (also in this issue) examined potential variables affecting measured VEGF levels prior to long term freezer storage, we focused on variables after collection as well as potential sources of error related to the measurement of urinary VEGF levels by enzyme linked immunosorbant assays (ELISA). We also chose to focus on potential causes of diminished reproducibility, including an evaluation of inter-assay precision, determined by the variation in VEGF sample results obtained from three different ELISA kit lot numbers and variations in the results obtained with identical ELISA lots when assays were performed by two lab technicians. We then examined variables after collection that had been suggested to affect levels of VEGF in urine biospecimens such as the time the samples were left at room temperature prior to assay, the addition of protease inhibitors prior to storage and the modification of the pH of the sample.

## Materials and methods

### Specimen collection

Human urine samples were collected from male and female adult patients receiving definitive radiation therapy for glioblastoma multiforme. In each case, samples were collected in accordance with approved protocols requiring informed consent. Patients were instructed to provide fresh, midstream urine specimens of at least 5 ml at three time points: (*i*) before receiving any radiation therapy, (*ii*) on the last day of radiation therapy and (*iii*) one month following completion of their therapy.

### Urine processing and VEGF analyses

After collection, urine specimens were divided into 4 ml aliquots and stored at –20°C. For each experiment, specimens were randomly selected from the cohort, thawed, divided into smaller aliquots for duplicate or triplicate analysis and stored at –20°C until analysis. VEGF levels were determined using a commercially available chemiluminescent ELISA kit (QuantiGlo®; R&D systems, Minneapolis, MN; http://www.rndsystems.com/pdf/qve00b.pdf) according to the manufacturer's instructions.

### Variation in VEGF levels between ELISA kit lot numbers

Eleven randomly selected samples were run in triplicate using three different ELISA kits with varying lot numbers (236856, 238697, 239328). The reproducibility of results obtained from the three kit lots was evaluated.

### Intra-technician reproducibility

Ten urine samples were randomly selected from the cohort. Two technicians, one with significant experience running the assay and the other with less experience, independently ran the 10 samples, in triplicate on the same plate. Measured VEGF levels were compared between the two technicians to determine intra-technician reproducibility.

### Time of thaw

Nine samples were randomly selected from the cohort to evaluate the effects of time of thaw. For each sample, aliquots were thawed at room temperature for five different periods of time before VEGF measurement: 1, 3, 5, 7 and 24 hrs.

### Evaluation of protease inhibition in urine samples

Thirteen randomly selected patient urine samples were divided into 200 μl aliquots and stored at -20°C overnight with or without the addition of a protease inhibitor. For samples with protease inhibitors, an appropriate mass of one mini, ethylenediaminetetraacetic acid (EDTA)-free tablet (Roche Applied Science, Mannheim, Germany) was added. Samples, stored at -20°C overnight, were run in triplicate following 3 and 24 hrs of sitting at room temperature on the lab bench. To confirm the protease inhibitor was not interfering with molecular activities of the ELISA assay prepared VEGF standards (0, 6.4, 32, 160, 800, 4000 and 10,000 pg/ml) were run in duplicate in the presence or absence of a protease inhibitor. Differences in relative light units were determined by luminometry.

### Effect of altering urine pH

The pH of four randomly selected patient urine samples was measured by a precision pH meter and microprobe (Accumet, Fischer Scientific) standardized for temperature. Alterations in pH were accomplished with the addition of 1N NaOH or 0.5N HCl. 200μl aliquots of samples were slowly titrated to pH 4, 5, 6 and 7 with the addition of the appropriate acid or base, stored overnight at -20°C, and analysed the next morning for the effect of pH on measured urinary VEGF levels.

### Statistical analyses

For each sample, a mean VEGF level and standard error were calculated. Statistical significance was determined using paired t-tests. Results were considered to be significant at *P* < 0.05. In addition, each experiment was performed in duplicate and the results were compared for further insight into the reproducibility of the assay.

## Results

### Effect of ELISA kit lot numbers on measured urinary VEGF levels

**Fig. 1:**
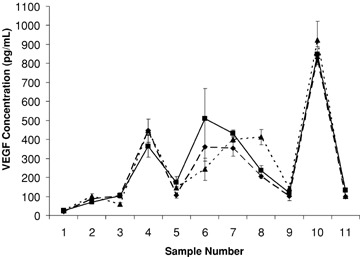
Inter-assay variation between three ELISA kit lot numbers. Of 11 samples, 9 showed less than 10% coefficient of variation across three lot numbers. Bars represent +/− S.E.M.

No significant variation was observed in urinary VEGF levels measured across three different lot numbers. The coefficients of variation (CVs) of 11 identical samples tested in triplicate ranged approximately 4–18%. Only three samples showed a CV of greater than 10% between lot numbers (Fig. 1 and Table 1). The samples with high variability had levels in the mid-range of observed values, well within the range of the standard curve generated from the kit supplied standards.

**Table 1:** Inter-assay precision (n = 3) between three lot numbers

Sample	Mean VEGF (pg/ml)	SEM	CV (%)
1	23.63	2.08	8.79
2	86.19	6.46	7.50
3	89.35	9.24	10.34
4	418.81	28.61	6.83
5	142.87	13.91	9.74
6	371.24	65.41	17.62
7	395.11	17.90	4.53
8	284.92	35.04	12.30
9	121.27	10.22	8.43
10	866.85	35.79	4.13
11	110.35	6.59	5.97

### Experience of technician improved the reproducibility of urinary VEGF measurements

**Fig. 2:**
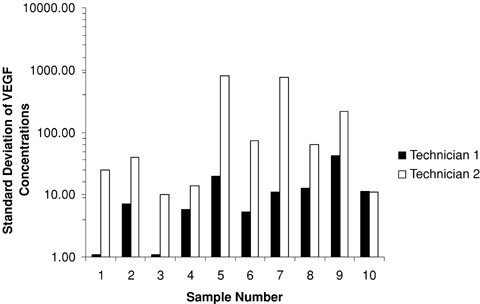
Differences in intra-assay variability for VEGF levels obtained from 10 samples. Technician 1 = more experienced technician.

To evaluate the reproducibility of the VEGF ELISA kit when the assay is performed by an experienced and an inexperienced laboratory technician, each technician ran triplicate samples in identical assay kits. Significant variations in VEGF concentrations were observed when VEGF levels were measured in ten identical samples by the two technicians (Fig. 2). For 9 of the 10 samples, the standard deviations of the experienced technician were lower than the standard deviations of the inexperienced technician (*P*= 0.041, paired t-test, one-tailed). In two replicate experiments, the more experienced technician again had a significantly higher reproducibility than the less experienced one.

### Effect of increasing time of thaw on measured urinary VEGF levels

**Fig. 3:**
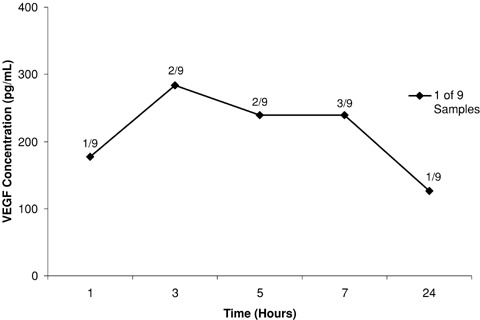
Urinary VEGF levels following 1, 3, 5, 7 and 24 hr thawing times. Urine samples attained their highest VEGF values between 3–7 hrs after removal from −20°C. Similar results were obtained from a second experiment.

We next evaluated the effect of the amount of time that elapses between removing the samples from the freezer and performing the ELISA assay by leaving the samples on the bench top for 1, 3, 5, 7 and 24 hrs before measurement of VEGF levels. Seven of nine samples attained their highest VEGF levels 3 to 7 hrs after being removed from the freezer and decreased at 24 hrs (Fig. 3).

### Effect of protease inhibitors on VEGF degradation between 3 and 24 hours

**Fig. 4:**
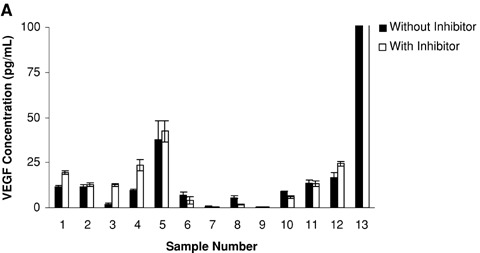

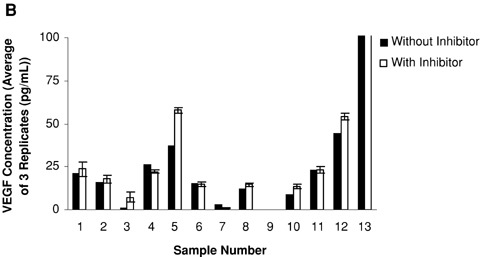
VEGF levels measured in the absence or presence of protease nhibitors after allowing urine to sit on the bench top for (**A**) 3 hr and (**B**) 24 hr.

To determine whether the reduction of VEGF levels between 3 and 24 hr was due to the presence of urinary proteases, VEGF levels in samples with and without protease inhibitors were compared following 3 and 24 hrs of sitting on the bench top (Fig. 4A and B). No significant difference was observed with or without the addition of protease inhibitors at either 3 or 24 hrs (*P*= 0.240 and 0.364, respectively; paired t-test, two-tailed) suggestin g that protein degradation over time was not influenced by urinary proteases. As an ELISA assay relies on enzymatic reactions between an antibody and substrate, we evaluated whether the protease inhibitor could interfere with the molecular activities of the kit. No difference was observed when the protease inhibitor was added to the standards used in the ELISA assay. Relative light units were similar between standards tested in the presence or absence of protease inhibitors (data not shown).

### Effect of altering urine pH on measured VEGF levels

**Fig. 5:**
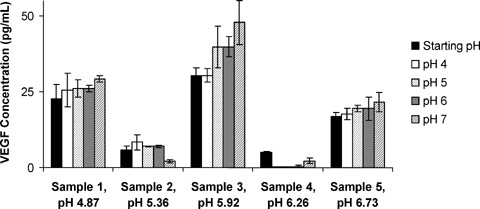
Changes in VEGF levels as a result of altering the pH of five samples.

In two experiments the pH measurements of five patient samples with initial pH values of 4.87, 5.36, 5.92, 6.26 and 6.73, were each altered to 4, 5, 6 and 7. No significant changes in VEGF levels were observed as a result of decreasing or increasing the pH of the urine samples (Fig. 5).

## Discussion

We investigated several potential variables that were suggested to affect the reproducibility and accuracy of the measurement of VEGF in urine samples obtained from patients receiving radiation treatment for glioblastoma multiforme. We suggested that differences in specimen characteristics, specimen processing and specimen storage may alter the results obtained. In addition to these specimen characteristics, we wished to evaluate the importance of technician experience and the use of ELISA kits from different lots.

As urine samples may be collected over a period of years, the inter-lot variability between human VEGF ELISA kits was of primary importance. Kits of different lot numbers may have different concentrations of antibody in coated wells, thereby altering the antigen-antibody binding efficiency between assays. However, our findings showed that the variability between lot numbers was low enough to justify using human VEGF-ELISA kits of differing lots for long-term prospective studies. CVs of the 11 identical samples tested in triplicate were in accordance with the CVs obtained by the manufacturer (range 4–10%). Thus, an inter-lot reproducibility of 85–90% can be expected.

Variations in VEGF ELISA results may also be dependent on the technician who is performing the experiment [17]. The results of this study showed that a more experienced technician may be able to perform the assay with higher reproducibility than a less experienced one. One possible solution to overcome this is the use of automated liquid handling, as pipetting variability is an obstacle to achieving intra-assay reproducibility in low-volume reactions.

Of additional importance is the time that elapses after urine biospecimens are removed from the freezer and left on the lab bench before assaying the biomolecules. As supersaturated urine specimens cool to ambient temperature, precipitation of calcium and phosphate, uric acid and proteins may occur. Additionally urine may contain bacterial growth, with accompanying proteoly-sis that can increase over time [18]. Therefore, it is best to standardize the time the sample spends at room temperature to minimize protein degradation or loss of protein *via* precipitation. Our results show that a thaw time of 3–7 hrs is optimal for obtaining maximal and consistent VEGF levels. For the purpose of our experiments, the sample thaw period was standardized to 3 hrs.

The addition of a protease inhibitor did not appear to overcome the instability of VEGF between 3 and 24 hrs, suggesting that VEGF degradation is not affected by the presence or absence of proteases within this time frame. While other authors have found that the addition of protease inhibitors enhances the recovery of urinary-associated proteins, this effect appears only when the inhibitor is added soon after collection [19].

Based on our findings, VEGF does not appear to be sensitive to neutral or acidic pH as its stability was maintained at a pH of 4, 5, 6 and 7. Similarly, Klasen *et al.*[20] did not find any relationship between pH and the urinary protein levels of albumin, transferrin and a1-microglobulin. In addition, the pH level of the urine did not affect the ability of the ELISA assay to detect VEGF levels, an important result as pH is known to alter the avidity of antibody-epitope interactions [21].

As more studies collect and archive urine samples to measure protein levels, it is important to assess the impact of urine preservation and storage methods on the levels of these molecules. Based on results reported here, urinary VEGF levels can be measured for long-term prospective studies since variation between ELISA kit lot numbers is insignificant. Intra-assay precision appears to be greater when a more experienced technician performs the assay. As a standard practice we suggest that, for prospective studies, the same technician perform the ELISA assay for each investigation. In addition, the time that the urine sample spends at room temperature on the bench top, between removal from the freezer and inclusion in the assay, should be standardized to 3–7 hrs. The addition of protease inhibitors during the sample thaw period does not appear to be essential to maintain optimal VEGF integrity. Additionally, the sample pH does not need to be standardized if samples are between a pH of 4 and 7.

While these variables have been evaluated for the measurement of VEGF levels in urine by ELISA, it is possible that these same variables may have different effects in other biospecimens and for different biomarkers. It is important to fully evaluate potential causes of measurement variability prior to initiating large studies to evaluate a biomarker. Finally, it is of critical importance to standardize storage and assay performance variables to ensure maximal reproducibility.
